# Effect of *APOB* polymorphism *rs562338 (G/A)* on serum proteome of coronary artery disease patients: a “proteogenomic” approach

**DOI:** 10.1038/s41598-021-02211-4

**Published:** 2021-11-23

**Authors:** Muneeza Zafar, Munazza Raza Mirza, Fazli Rabbi Awan, Muhammad Tahir, Rabia Sultan, Misbah Hussain, Ahmed Bilal, Shahid Abbas, Martin R. Larsen, Muhammad Iqbal Choudhary, Imran Riaz Malik

**Affiliations:** 1 Dr. Panjwani Center for Molecular Medicine and Drug Research, International Center for Chemical and Biological Sciences ICCBS), University of Karachi, Karachi, 75270 Pakistan; 2 Department of Biotechnology, University of Sargodha, Sargodha, Pakistan; 3 Diabetes and Cardio-Metabolic Disorders Lab, Health Biotechnology Division, National Institute for Biotechnology and Genetic Engineering (NIBGE), Jhang Road, P.O. Box. 577, Faisalabad, Pakistan; 4 Allied Hospital, Faisalabad Medical University, Faisalabad, Pakistan; 5 Faisalabad Institute of Cardiology (FIC), Faisalabad, Pakistan; 6 Department of Biochemistry and Molecular Biology, University of Southern Denmark, Odense, Denmark

**Keywords:** Biotechnology, Molecular biology

## Abstract

In the current study, *APOB* (*rs1052031)* genotype-guided proteomic analysis was performed in a cohort of Pakistani population. A total of 700 study subjects, including Coronary Artery Disease (CAD) patients (n = 480) and healthy individuals (n = 220) as a control group were included in the study. Genotyping was carried out by using tetra primer-amplification refractory mutation system-based polymerase chain reaction (T-ARMS-PCR) whereas mass spectrometry (Orbitrap MS) was used for label free quantification of serum samples. Genotypic frequency of GG genotype was found to be 90.1%, while 6.4% was for GA genotype and 3.5% was for AA genotypes in CAD patients. In the control group, 87.2% healthy subjects were found to have GG genotype, 11.8% had GA genotype, and 0.9% were with AA genotypes. Significant (*p* = 0.007) difference was observed between genotypic frequencies in the patients and the control group. The rare allele AA was found to be strongly associated with the CAD [OR: 4 (1.9–16.7)], as compared to the control group in recessive genetic model (*p* = 0.04). Using label free proteomics, altered expression of 60 significant proteins was observed. Enrichment analysis of these protein showed higher number of up-regulated pathways, including phosphatidylcholine-sterol *O*-acyltransferase activator activity, cholesterol transfer activity, and sterol transfer activity in AA genotype of *rs562338* (G>A) as compared to the wild type GG genotype. This study provides a deeper insight into CAD pathobiology with reference to proteogenomics, and proving this approach as a good platform for identifying the novel proteins and signaling pathways in relation to cardiovascular diseases.

## Introduction

Coronary artery disease (CAD) refers to the chronic inflammation due to gradual accumulation of lipids and fibrous elements over a life-span that leads to atherosclerotic plaque formation in arteries of heart. Multiple genetic risk variants, polygenic traits, and exposure to atherogenic environment are suggested to be involved in CAD manifestation^1,2^. Besides, individuals with normal low density lipoprotein (LDL)-cholesterol may also develop atherosclerosis without any conventional risk factors. Therefore, a better understanding of the disease etiology, and efficient therapeutic interventions are mandatory. It is now evident that genetics or heritability may explain ≈ 25% of the phenotypic variance in CAD^2,3^. Several genome-wide association studies (GWAS) have led to the identification of many genetic risk factors including > 300 chromosomal loci which are significantly associated with CAD risk^4^.

Apolipoprotein B gene (*APOB*) is one of the lipid associated genetic factors located on human chromosome 2. Currently, it has become a research hot spot due to its vital importance in the lipoprotein metabolism^5–8^. In humans, apolipoprotein B acts as a lipid transporter, and found as a structural component of all non-HDL lipoproteins. It has two isoforms, ApoB100 and ApoB48. ApoB100 is the major isoform of ApoB synthesized in hepatocytes, and found in Lp(a) LDL, VLDL, and VLDL remnants. The second isoform apoB48 (the truncated form) is synthesized in the intestinal enterocytes, and is the structural protein of chylomicrons and chylomicron remnants. Furthermore, ApoB100 contains the ligand that binds to the LDL receptor on the surface of hepatocytes, and other body cells, and is involved in the cholesterol rich lipoprotein metabolism^9^. Recent studies have shown that ApoB concentration provides direct measure of the total lipoproteins (LDL, LDL remanats, Lp(a)) that leads to atherosclerosis, as every single atherogenic particle comprised of one molecule of ApoB inside^9^. It is also a well-known risk factor for hyperlipidemia, and its deficiency leads to hypobetalipoproteinemia^10,11^. Genetic association studies have indicated the association of dyslipidemia with multiple single nucleotide polymorphisms (SNPs) present in *APOB*^12,13^. Furthermore, according to the European Society of Cardiology (ESC), and European Atherosclerosis Society (EAS), during the atherosclerotic cardiovascular disease (ASCVD) risk assessment, ApoB evaluation is preferred over the measurement of LDL-cholesterol in patients with hypertriglyceridemia, obesity, and diabetes, as it provides a better and direct measure of atherosclerosis^14^.

About 90% of the disease associated with genetic risk variants have modest effect sizes, and are located outside the protein-coding region^15,16^. Such variants can explain only ∼ 25% of the overall disease heritability. This suggests that genetic variations may contribute to disease risk in complex, interactive, and non-linear way^17^. This may also leads to pathological changes in diverse cell types at the tissue, organ, or at molecular levels^18^.

In the current era of technological advances, the breadth of available Omics data has expanded exponentially^17^. With the emergence of high-throughput Omics platforms, it is now possible to have a deep insight at a molecular level in the disease mechanism, etiology, and pathophysiology at different stages and at different stages of the disease. Moreover, the discovery, selection, and reliability of candidate biomarkers for the detection of a disease before its onset, and progression, is now easier and more reliable through these advanced analytical technologies. The proteomic studies enable us to identify the potential genes asserted by proteins^18^. These proteins may be acting as catalysts of enzymatic reactions in the molecular signaling cascades, and protein–protein physical interactions^19^. At the same time, it has been established that no single type of data can fully explain the manifestation of complex disease phenotypes^20,21^. Rather, it requires multiple layers of information across several omics domains in order to integrate data for more precise characterization of phenotypes^22^. Recently, proteogenomics, the integration of genomics and proteomics data has become very popular for identifying novel proteins or signaling pathways in relation to various diseases^23^.

Here, the present study was designed to investigate the association of genetic determinant (*rs562338*) of *APOB* gene with the risk of CAD in local population. In the second step, the *APOB* guided proteomics was applied by combining both genotyping and LC/MS based proteomic data to identify underlying molecular mechanisms for better understanding of the complex pathology of CAD.

## Material and methods

### Subject selection

Samples from the study subjects were collected between Jan-Nov 2016. The overall analysis including biochemical, genetic, proteomics, and data acquisition duration was between 2016 and 2020. The methods were performed according to the relevant guidelines and regulations (National Institute for Biotechnology and Genetic Engineering, Faisalabad, Pakistan ethics review committee). After obtaining informed consent, 480 CAD patients (aged 35–65 years) were included in the study from the Allied Hospital and Faisalabad Institute of Cardiology (FIC), Faisalabad, Pakistan. All patients had undergone coronary catheterization, and were examined through electrocardiogram (ECG) by expert cardiologists. Questionnaires including demographic details, disease diagnosis, history of smoking habits, family history of disease, and dietary routines were documented for all the study subjects. Individuals with any chronic medical conditions, such as diabetes history, acute myocardial infraction (MI) within past 3 months, unstable angina, significant valvular heart disease, and having serum creatinine level ˃ 3.0 were excluded from the study. The healthy control group was formed with 220 individuals with no history of any metabolic disease like hypertension, diabetes, CAD, stroke etc. They never went through coronary catheterization and were not taking any medicine, such as anticoagulants, platelet inhibitors, antihypertensive, and cholesterol lowering therapy. According to the American Heart Association guidelines, hypertension was defined as arterial blood pressure > 140/90 mm Hg and measured in sitting position after 5 min of rest. Body mass index (BMI) was calculated as weight in kilogram, and height in meter per square (kg/m^2^), and waist circumference was measured in standing position with a measuring tape midway between last rib and top of coxal bones, while hip circumference was measured from the widest part of the hip to calculate waist-to-hip ratio (WHR)^24,25^.

### Laboratory assessment and serological testing

Using standard venipuncture, 2–5 mL blood was drawn from both the healthy control and CAD patients. Serum was separated by centrifugation at 1000×*g* for 10 min using automated centrifuge (Thermo Fisher Scientific USA). Biochemical analysis and serological testing was carried out on a semi-automated clinical chemistry analyzer (MicroLab300 (Merck, USA). Subjects with serum protein abnormalities, such as elevated liver function test, renal insufficiency, and uncontrolled diabetes, were excluded. Serum samples were labeled accordingly, and stored at − 20 °C.

### Genotyping of *APOB rs562338 (G/A)*

Genomic DNA was isolated from each sample by standard phenol: chloroform method^26^. For genotyping, tetra primer-amplification refractory mutation system-polymerase chain reaction (T-ARMS-PCR) was optimized^27^ to detect G/A polymorphism of *APOB* (*rs562338*), using two pairs of primers in a single reaction mixture^27^.

- Pair-1 sense: 5′-CATTATTGCTGATGATAGGCATGATGTTG-3′
  Atisense: 3′-CATGGTTTGCATACATCACATTTTCTTTAACC-5′,
- Pair-2 sense: 5′-CTAAATGTTCATTGTCTTGACAGATGAATTCA-3′
  Antisense: 3′-CTGGGTGCACAGTTGGATTTGAACAGG-5′.

The resultant three genotypes were GG (381 bp), AA (354 bp), and for GA both bands were present along with the control band of 672 bp size. PCR amplification conditions were: 95 °C for 5 min, 35 cycles of amplification at 95 °C for 1 min, 64.3 °C for 1 min, 72 °C for 30 s, and a final extension at 72 °C for 10 min.

### Protein estimation and tryptic digestion

On the basis of genotyping results, serum of each genotype carrying sample were pooled together by taking 2 µL from each subject, and categorized as GG, GA, and AA within disease group. Protein estimation was carried out by using standard Bradford assay^28^. For tryptic digest, 80 μL of 1 M NH_4_HCO_3_ was added to 100 µg of estimated protein to adjust the pH to 8–8.5. For denaturation, 20 μL of 40 mM nOGP (n-octyl glucopyranoside), and 50 μL of 45 mM DTT (dithiothreitol) were added, followed by incubation at 90 °C for 30 min with 800 rpm on a thermomixer (Eppendorf AG, Germany). After denaturation, the protein samples were cooled to room temperature, and then alkylated by adding 50 μL of 100 mM iodoacetamide (final concentration 20 mM). A second round of incubation was performed in the dark at room temperature for 15 min. After incubation, 1400 μL of deionized water was added, followed by addition of 10 μg trypsin (10 μL of 1 μg/μL). For protein digestion, a third round of overnight incubation on thermomixer at 37 °C was carried out. Lastly, digestion was stopped by adding 60 μL of 2% TFA (pH ≤ 3), and all digests were stored at − 20 °C^29^. For desalting of peptides, reverse phase cartridges ZIP TIP C18 (Millipore Corporation, USA) were used according to the manufacturer's instructions. Each sample was evaporated using a Speedvac (Thermo Scientific U.S.A.), and subsequently reconstituted in 0.1% TFA. All samples were quantified prior to proceeding for mass spectrometry analysis using Qubit reagent (Thermo Scientific, USA).

### Mass spectrometry analysis

The peptide mixtures were analyzed by nano-LC–MS/MS on an Orbitrap Q-Exactive HF-X (Thermo Fisher Scientific USA) coupled to an EASY-LC 1000 system (Thermo Fisher Scientific, USA). The data was generated by using previously described protocol^30^.

### Protein quantification and data analysis

The raw files generated on Orbitrap Q-Exactive HF-X were used to generate the protein profile on MaxQuant (v2.3.2, Matrix Science, UK) using Andromeda search engine with default search settings^31^. The discovery rate was set as 1%. The spectra were searched against *Homo sapiens* proteins in the UniProt/Swiss-Prot database (http://www.uniprot.org/). During the main search, the mass tolerances for precursor and fragment ions were set to 4.5 and 20 ppm. Enzyme specificity was set as carboxy-terminal to arginine and lysine (trypsin) and maximum two missed cleavages were allowed at arginine/lysine–proline bonds. Carbamidomethylation of cysteine residues was set as a fixed modification, and variable modifications were set as oxidation of methionine (to sulfoxides), and acetylation of protein amino-termini. Proteins were quantified by the MaxLFQ algorithm, integrated in the MaxQuant software. Only proteins with at least one unique or razor peptide were retained for identification, while a minimum ratio count of two unique or razor peptides was required for quantification^32,33^.

### Overrepresentation analysis

Significance of differential protein levels between healthy control and CAD patients group was established using t-test. *P-*values were corrected for multiple testing according to Benjamini and Hochberg^34^. Significance of differential protein levels was assessed while adhering to a 10% FDR cutoff. To visualize the expression trend in different genotypes, a heat map was generated by using Perseus software (v.1.6.10.50). For overrepresentation analysis of molecular functioning, one sided Fisher’s exact test was used with significance at an alpha-level of 0.05. Pathway analysis was performed using Reactome Pathway Analysis (https://reactome.org/).

### String analysis

The STRING (Search Tool for the Retrieval of Interacting Genes/Proteins) was used for critical assessment, and integration of protein–protein interactions (http://string-db.org/). The interactions were drawn from the experimental evidence as well as predictions based on knowledge gained from other organisms^35^. By using STRING, prioritized significant proteins were mapped, and a network image was created.

### Statistical analysis

Data are presented as mean ± standard deviation for continuous variables, and for categorical variables, expressed in frequency, and percentage. To analyze the significant effect of biochemical parameters on both the disease, and the control groups, univariate general linear model (ANCOVA) adjusted for age and gender was applied. Chi-square test and multinomial regression model adjusted for age and gender was used to measure odds ratio (95% CI). Allelic and genotypic frequencies were calculated by direct gene counting method, and Hardy–Weinberg Equilibrium was measured. All these statistical analyses were performed on SPSS (IBM SPSS 20).

### Compliance with ethical standards

All procedures performed in this study involving human participants were approved by the National Institute for Biotechnology and Genetic Engineering, Faisalabad, Pakistan) ethics review committee. Informed consent was obtained from all the study participants.

## Results

**Figure 1:**
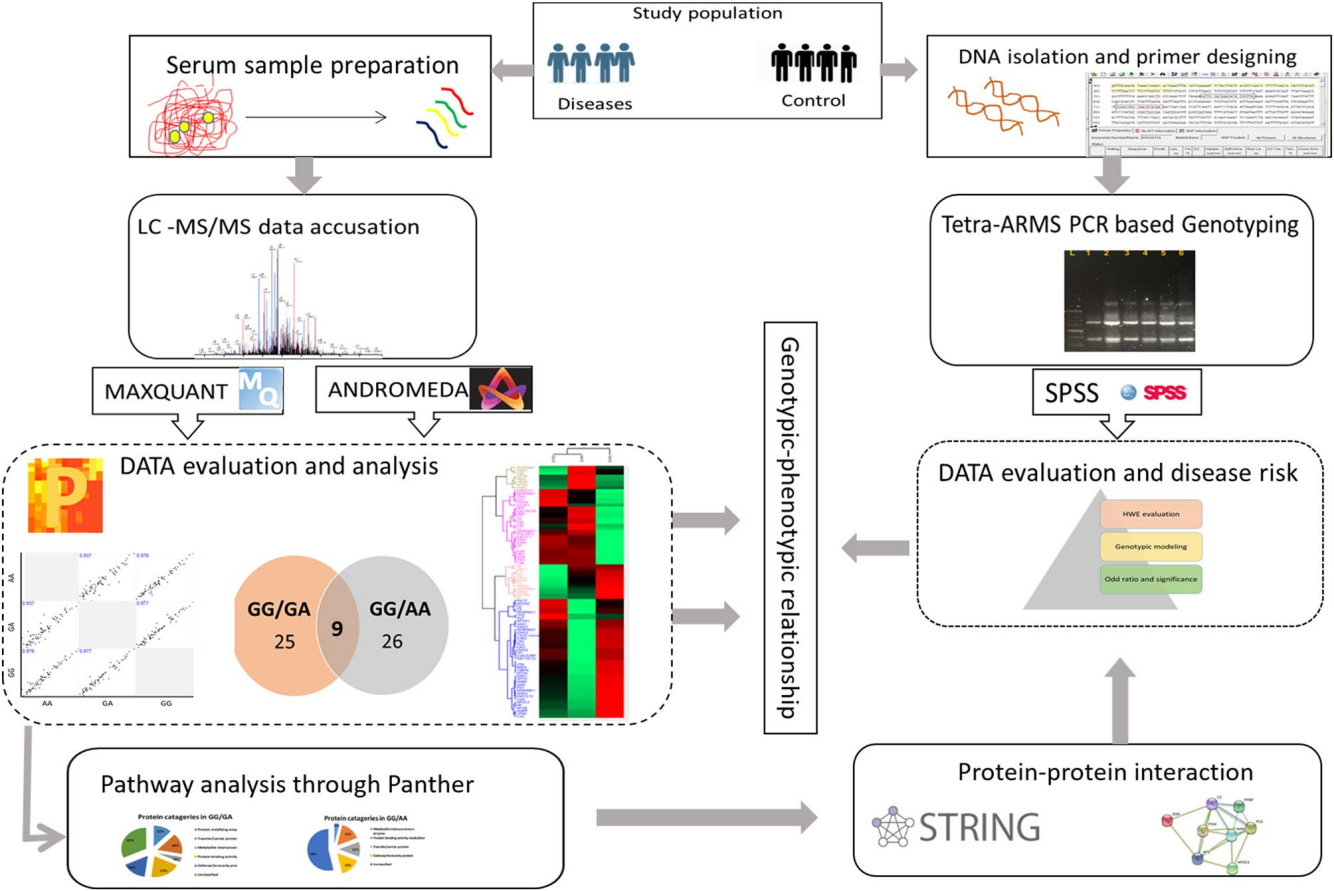
Schematic representation of *rs562338* genotyping and its genotype based differential expression of proteins by using label-free quantitative (LFQ) proteomics. The shotgun proteomics study with disease-control design (n = 700) was conducted to dissect comparative differential serum proteome with genetic variant. A genotypic-phenotypic relation was studied. Further details can be found under Experimental Procedures.

**Figure 2:**
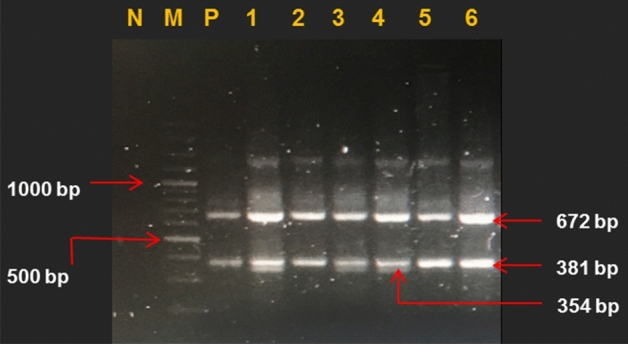
T-ARMS PCR assay based genotyping of *APOB* rs*562338* (*G/A*) polymorphism on 2.5% agarose gel (cropped image). Lane M shows 1000 bp molecular marker, lane N shows negative control and lane P for positive control. Lanes 2, 5 and 6 show GG genotype of 381 bp, along with control band of 672 bp. Lanes 1, 3 and 4 represent GA genotype with both bands of 351 bp and 381 bp, along with control of 672 bp (Full length figure is provided in Supplementary Fig. S2).

The overall workflow of the research design is presented in Fig. 1. Initially, baseline clinical and anthropometric parameters were measured in both the disease, and control groups (Table 1). Among all study subjects, 80% subjects were male, and 20% were female in the control group, while 45% were male and, 55% were female in the disease group. Approximately 9% of the selected subjects with CAD were taking antihypertensive drugs and < 20% were prescribed to take statins (cholesterol lowering drug). The samples were collected at the time of admission or after 1 day of their admission. Anthropometric and clinical parameters such as BMI, WHR, blood glucose, and blood pressure were significantly higher (*p* < 0.05) in the disease group when compared to the control group. Furthermore, a significant difference was found in the total cholesterol, HDL-C, LDL-C, triglycerides, serum uric acid, and serum creatinine in both the disease, and control groups. Genotyping was performed by T-ARMS PCR, as shown in Fig. 2 (full image of the gel is provided in Supplementary Fig. S2). By use of the gene counting method, both genotypic and allelic frequencies of rs*562338* (G/A) were calculated (Table 2). In the CAD group, 90.1% were found to have GG, 6.4% were GA, and 3.5% were having AA genotypes. In the control group, 87.2% had GG, 11.8% were having GA and, 0.9% had AA genotypes. In the present study, genotypic frequencies were within the Hardy–Weinberg equilibrium (HWE) (χ^2^ = 1.07, *p* = 0.29). Significant differences were observed between genotypic frequencies (*p* = 0.007) of both the disease, and the control groups, while their allelic frequencies showed non-significant differences. Furthermore, in genetic modeling, the rare allele (A) was found to be strongly associated with CAD [OR = 4 (1.9–16.7)] when compared to the control subjects in recessive genetic model (*p* = 0.04).

**Table 1:** Baseline anthropometric, clinical, and biochemical parameters of study subjects.

Biochemical parameters	Control group n = 220	Disease group n = 480	*p-*value
**Age**	53.24 ± 11.1	54.54 ± 10	0.130
**Male**	183 (83%)	270 (56%)	≤ 0.001**
**Female**	37 (17%)	210 (44%)	
**BMI (kg/m**^**2**^**)**	22.4 ± 6.35	25 ± 3.2	≤ 0.001**
**WHR (mean)**	0.86 ± 0.09	1.00 ± 0.13	≤ 0.001**
**Glucose (mg/dL)**	99.8 ± 12	145.81 ± 12	≤ 0.001**
**Systolic (mmHg)**	119.47 ± 15	137.29 ± 26	≤ 0.001**
**Diastolic (mmHg)**	79.22 ± 11	86.9 ± 15	≤ 0.001**
**Serum total cholesterol (mg/dL)**	187.8 ± 6.3	192.1 ± 8.5	≤ 0.001**
**Serum LDL-C (mg/dL)**	83.6 ± 11.9	112.5 ± 54.4	≤ 0.001**
**Serum HDL-C (mg/dL)**	48.6 ± 8.0	40.8 ± 13.7	≤ 0.001**
**Serum TG (mg/dL)**	248.1 ± 116.1	205.1 ± 84.9	≤ 0.001**
**Creatinine (mg/dL)**	0.89 ± 0.24	1.15 ± 0.79	≤ 0.001**
**Uric acid (mg/dL)**	6.32 ± 1.34	6.74 ± 2.4	0.016
**Serum albumin (g/dL)**	4.09 ± 0.31	4.41 ± 1.31	0.564
**Medication**	No	Yes	
Antihypertensive (ACE inhibitors/ARBs)	–	43 (9%)	–
Lipid lowering (Statins)	–	67 (14%)	–

*BMI* body mass index, *WHR* waist to hip ratio, *LDL-C* low-density lipoprotein cholesterol, *HDL-C* high-density lipoprotein cholesterol, *TG* triglycerides; Diabetes was defined as fasting blood glucose of ≥ 126 mg/dl. *ARBs* angiotensin II receptor blockers, *ACE* (angiotensin-converting-enzyme). Values are expressed as mean ± SD, and percent (frequency). Univariate General linear model (ANCOVA) adjusted for age and gender was used to analyze the effect of different variables in study groups. **Significant (*p-*value < 0.01).

**Table 2:** Genotypic, allelic frequencies and odds ratio of *APOB* rs*562338* (*G/A*) polymorphism in control and patient groups.

Gene	*rs562338*(*G/A*) polymorphism	Frequencies (%)		Association		OR (95% CI)
		Disease (n = 480)	Control (n = 480)	χ^2^	*p-*value	
*APOB*	Genotypes	GG: 431 (90.1)	GG: 192 (87.2)			
		GA: 30 (6.4)	GA: 26 (11.8)	5.8	0.01*	–
		AA: 19 (3.5)	AA: 2 (0.9)	4.3	0.03*	
	Allelic model	G: 892 (93)	G: 410 (93)			1.58 (0.6–1.6)
		A: 68 (7)	A: 30 (7)	0.36	0.9	
	Dominant model	GG: 431 (90) GA + AA: 49 (10)	GG: 192 (87) GA + AA: 28 (13)	1.0	0.29	0.74 (0.45–1.22)
	Recessive model	GG + GA: 461 (96) AA: 19 (4)	GG + GA: 218 (99.1) AA: 2 (0.9)	4.8	0.04*	4 (1.9–16.7)
	Codominance model	GG + GA: 461 (90) GA + AA: 49 (10)	GG + GA: 218 (89) GA + AA: 28 (11)	0.57	0.32	0.86 (0.5–1.3)

*Significant (*p***-**value ≤ 0.05).

**Figure 3:**
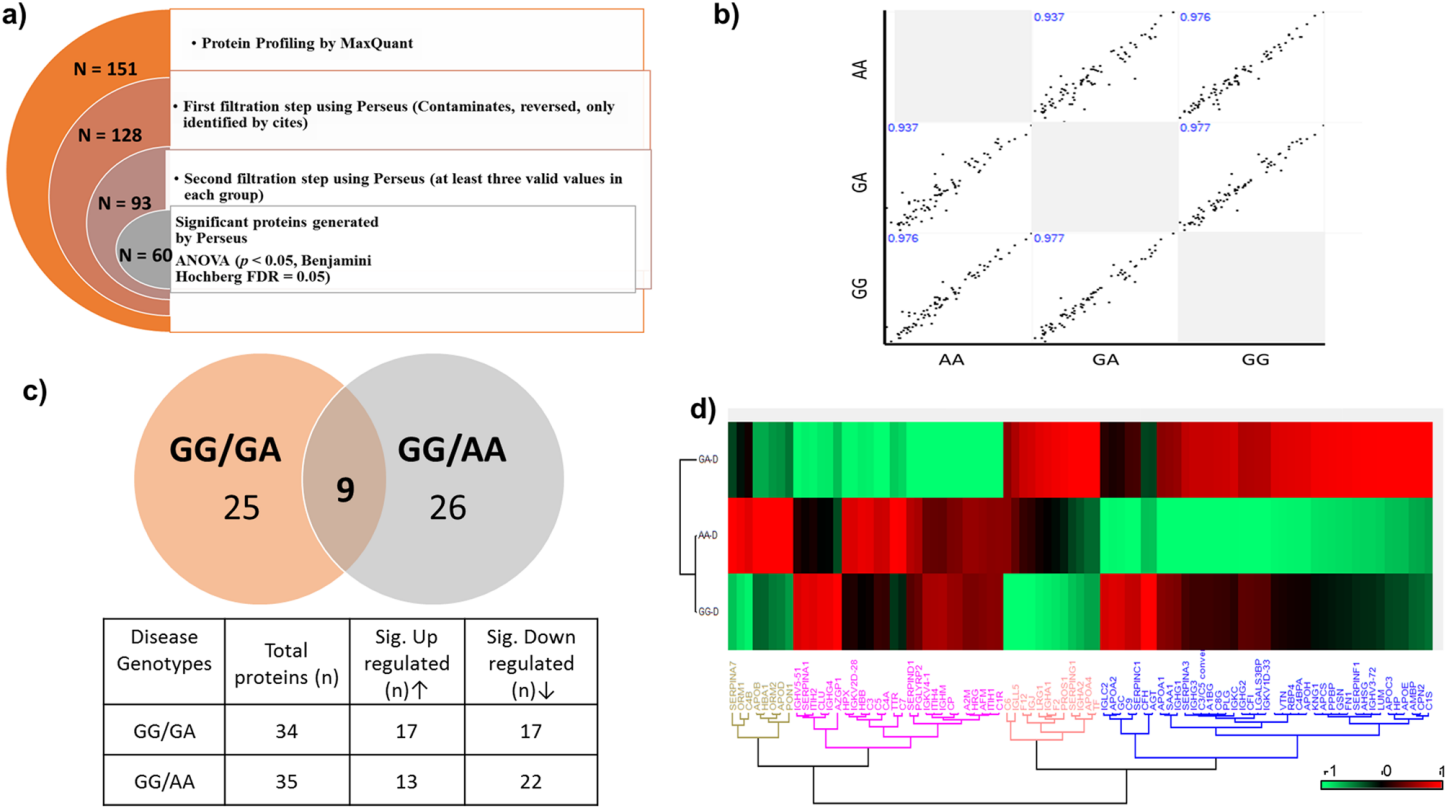
Schematic representation of proteomic data analysis. (**a**) Filtration steps applied to the identified proteins. (**b**) Scatter plot showing reproducibility of proteins in each genotype. (**c**) Venn diagram depicting significant proteins distribution and table represent all proteins with relevant up-regulated and down regulated pattern in both genotypes. (**d**) Heat map of significant proteins in both genotypes.

**Figure 4:**
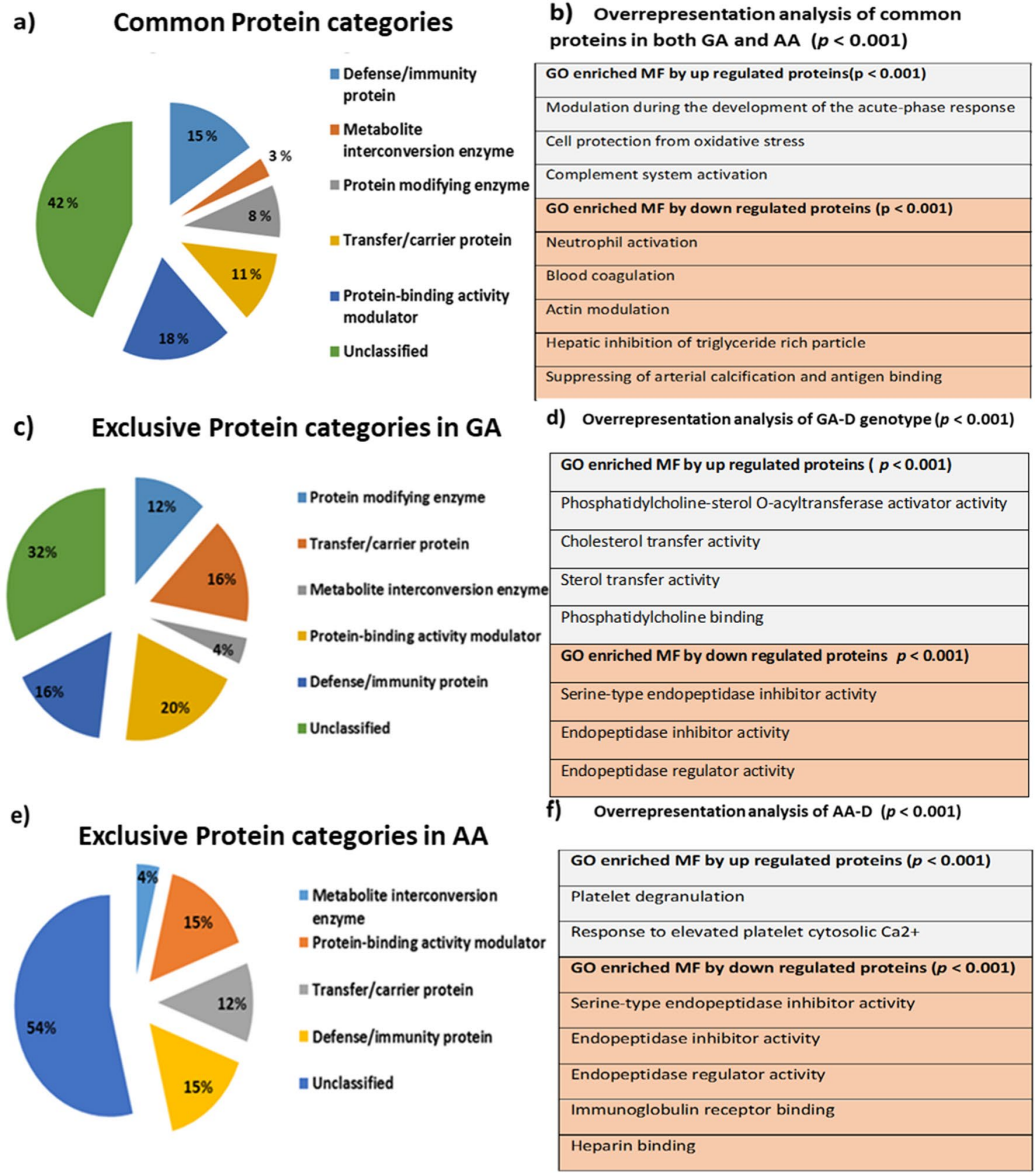
Distribution of both common and exclusive protein categories and their respective overrepresentation analysis. (**a**) Common Protein categories found in both AA and GA genotype carriers as compared to reference GG genotype. (**b**) Over representation analysis of differential up (grey) and down (pink) GO annotations of common proteins. (**c**) Exclusive Protein categories found in GA genotype. (b) Overrepresentation analysis of exclusively up (grey) and down (pink) GO annotations in GA genotype. (**c**) Exclusive Protein categories found in AA genotype. (**d**) Overrepresentation analysis of exclusively up (grey), and down (pink) GO annotations in AA genotype.

For the proteomic analysis, pooled serum samples of each genotype were analyzed on Q-Exactive HF-X. Each sample was run in triplicate. A total of 151 proteins were identified in all samples using MaxQuant (A list of these proteins is provided in Supplementary Table S1). By applying different filtering steps in Perseus (v.1.6.10.50), including removing contaminates, reversed and only identified by site, a total of 60 significant proteins were obtained, as depicted in Fig. 3a. Reproducibility of these significant proteins between the genotypes was ensured by using multiscatter plot using correlation coefficient (Fig. 3b). The data was further analyzed for disease group to check the differences between subjects having GA, and AA genotype with the normal genotype GG. Out of total 60 significant proteins, 25 were exclusively identified in GA genotype, and 26 were exclusively identified in AA genotype (Fig. 3c), while 9 proteins were found to be common in both the genotypes. A heat map of all proteins is presented Fig. 3d, depicting the fold differences of XIC intensities in both GA and AA genotypes. Detailed description of each protein with FC (log^2^) and *p-*values are listed in Table 3. Out of these 9 common proteins, three proteins ITIH4, HPX, and C3, showed higher differential expressions in the AA genotype as compared to the GA in comparison to the wild type GG genotype (Table 3). Gene Ontology (GO) of protein molecular functions with relevance to CAD showed that these proteins were involved in the modulation of cell migration and proliferation during development of acute-phase response, cell protection from oxidative stress, and complement system activation (Fig. 4a,b).

**Table 3:** Genotype (rs*562338*) based differential expression of serum proteins in CAD patients.

Proteins IDs	Protein name	Gene name	Unique peptide	Disease genotypes GG vs. GA GG vs. AA			
				FC(log^2^)	*p-*value	FC(log^2^)	*p*-value
Q14624-3	Inter-alpha-trypsin inhibitor heavy chain H4	ITIH4	34	0.930**↓**	0.000	1.032**↑**	0.002
P02790	Hemopexin	HPX	24	0.936**↓**	0.000	1.032**↑**	0.003
P01024	Complement C3	C3	138	0.940**↓**	0.000	1.057**↑**	0.000
P02775	Platelet basic protein	PPBP	5	1.035**↑**	0.002	0.973**↓**	0.005
P00747	Plasminogen	PLG	31	1.056**↑**	0.020	0.987**↓**	0.035
P06396-2	Gelsolin	GSN	13	1.014**↑**	0.000	0.978**↓**	0.010
P02656	Apolipoprotein C-III	APOC3	5	1.022**↑**	0.008	0.984**↓**	0.027
C9JV77	Alpha-2-HS-glycoprotein	AHSG	14	1.004**↑**	0.001	0.980**↓**	0.011
P04433	Immunoglobulin kappa variable 3–11	IGKV3-11	3	1.001**↑**	0.012	0.969**↓**	0.003
P09871	Complement C1*s* subcomponent	C1S	8	1.023**↑**	0.009	–	–
P02652	Apolipoprotein A-II	APOA2	11	1.018**↑**	0.020	–	–
P00738	Haptoglobin	HP	19	1.027**↑**	0.006	–	–
P02751-14	Fibronectin	FN1	51	1.015**↑**	0.036	–	–
P07360	Complement component C8 gamma chain	C8G	5	1.034**↑**	0.002	–	–
A0A3B3ISJ1	Vitamin K-dependent protein S	PROS1	11	1.022**↑**	0.013	–	–
P06727	Apolipoprotein A-IV	APOA4	32	1.016**↑**	0.025	–	–
P00734	Prothrombin	F2	22	1.019**↑**	0.013	–	–
A0A0G2JMB2	Immunoglobulin heavy constant alpha 2	IGHA2	1	1.021**↑**	0.009	–	–
P13671	Complement component C6	C6	13	1.021**↑**	0.013	–	–
P02787	Serotransferrin	TrF	73	1.029**↑**	0.004		
P02766	Transthyretin	TTR	11	–	–	1.022**↑**	0.005
P69905	Hemoglobin subunit alpha	HBA1	6	–	–	1.064**↑**	0.000
P19652	Alpha-1-acid glycoprotein 2	ORM2	4	–	–	1.024**↑**	0.004
P04114	Apolipoprotein B-100	APOB	158	–	–	1.030**↑**	0.002
P05090	Apolipoprotein D	APOD	6	–	–	1.034**↑**	0.003
P05543	Thyroxine-binding globulin	SERPINA7	7	–	–	1.017**↑**	0.013
P27169	Serum paraoxonase/arylesterase 1	PON1	13	–	–	1.030**↑**	0.003
P02763	Alpha-1-acid glycoprotein 1	ORM1	9	–	–	1.035**↑**	0.002
P13671	Complement component C6	C6	13	–	–	1.021**↑**	0.013
P02787	Serotransferrin	TF	73	–	–	1.018**↑**	0.020
P05546	Heparin cofactor 2	SERPIND1	14	0.97**↓**	0.002	–	–
P06312	Immunoglobulin kappa variable 4–1	IGKV4-1	2	0.96**↓**	0.001	–	–
P36955	Pigment epithelium-derived factor	SERPINF1	13	0.98**↓**	0.005	–	–
P00450	Ceruloplasmin	CP	55	0.98**↓**	0.009	–	–
P25311	Zinc-alpha-2-glycoprotein	AZGP1	16	0.98**↓**	0.006	–	–
A0A0C4DH38	Immunoglobulin heavy variable 5–51	IGHV5-51	2	0.95**↓**	0.001	–	–
Q5T985	Inter-alpha-trypsin inhibitor heavy chain H2	ITIH2	26	0.98**↓**	0.005	–	–
A0A3B3ISR2	Complement subcomponent C1r	C1R	13	0.98**↓**	0.013	–	–
P01009	Alpha-1-antitrypsin	SERPINA1	2	0.98**↓**	0.003	–	–
P01861	Immunoglobulin heavy constant gamma 4	IGHG4	6	0.97**↓**	0.003	–	–
P04196	Histidine-rich glycoprotein	HRG	16	0.97**↓**	0.002	–	–
P43652	Afamin	AFM	18	0.97**↓**	0.002	–	–
P01023	Alpha-2-macroglobulin	A2M	92	0.95**↓**	0.000	–	–
P68871	Hemoglobin subunit beta	HBB	10	0.99**↓**	0.030	–	–
P05546	Heparin cofactor 2	SERPIND1	14	–	–	0.99**↓**	0.034
P06312	Immunoglobulin kappa variable 4–1	IGKV4-1	2	–	–	0.98**↓**	0.026
P36955	Pigment epithelium-derived factor	SERPINF1	13	–	–	0.95**↓**	0.000
P01008	Antithrombin-III	SERPINC1	15	–	–	0.97**↓**	0.004
P02743	Serum amyloid P-component	APCS	6	–	–	0.98**↓**	0.006
Q08380	Galectin-3-binding protein	LGALS3BP	9	–	–	0.98**↓**	0.013
P0DJI8	Serum amyloid A-1 protein	SAA1	2	–	–	0.98**↓**	0.010
P01834	Immunoglobulin kappa constant	IGKC	20	–	–	0.99**↓**	0.026
A0A0A0MS08	Immunoglobulin heavy constant gamma 1	IGHG1	16	–	–	0.92**↓**	0.000
P01042-2	Kininogen-1	KNG1	1	–	–	0.98**↓**	0.010
P01859	Immunoglobulin heavy constant gamma 2	IGHG2	8	–	–	0.99**↓**	0.031
P02760	Protein AMBP	AMBP	7	–	–	0.98**↓**	0.008
P0DOY2	Immunoglobulin lambda constant 2	IGLC2	6	–	–	0.98**↓**	0.004
A0A4W8ZXM2	Immunoglobulin heavy variable 3–72	IGHV3-72	1	–	–	0.97**↓**	0.002
A0A2Q2TTZ9	Immunoglobulin kappa variable 1–33	IGKV1D-33	2	–	–	0.98**↓**	0.021
P08603	Complement factor H	CFH	39	–	–	0.99**↓**	0.036

**Figure 5:**
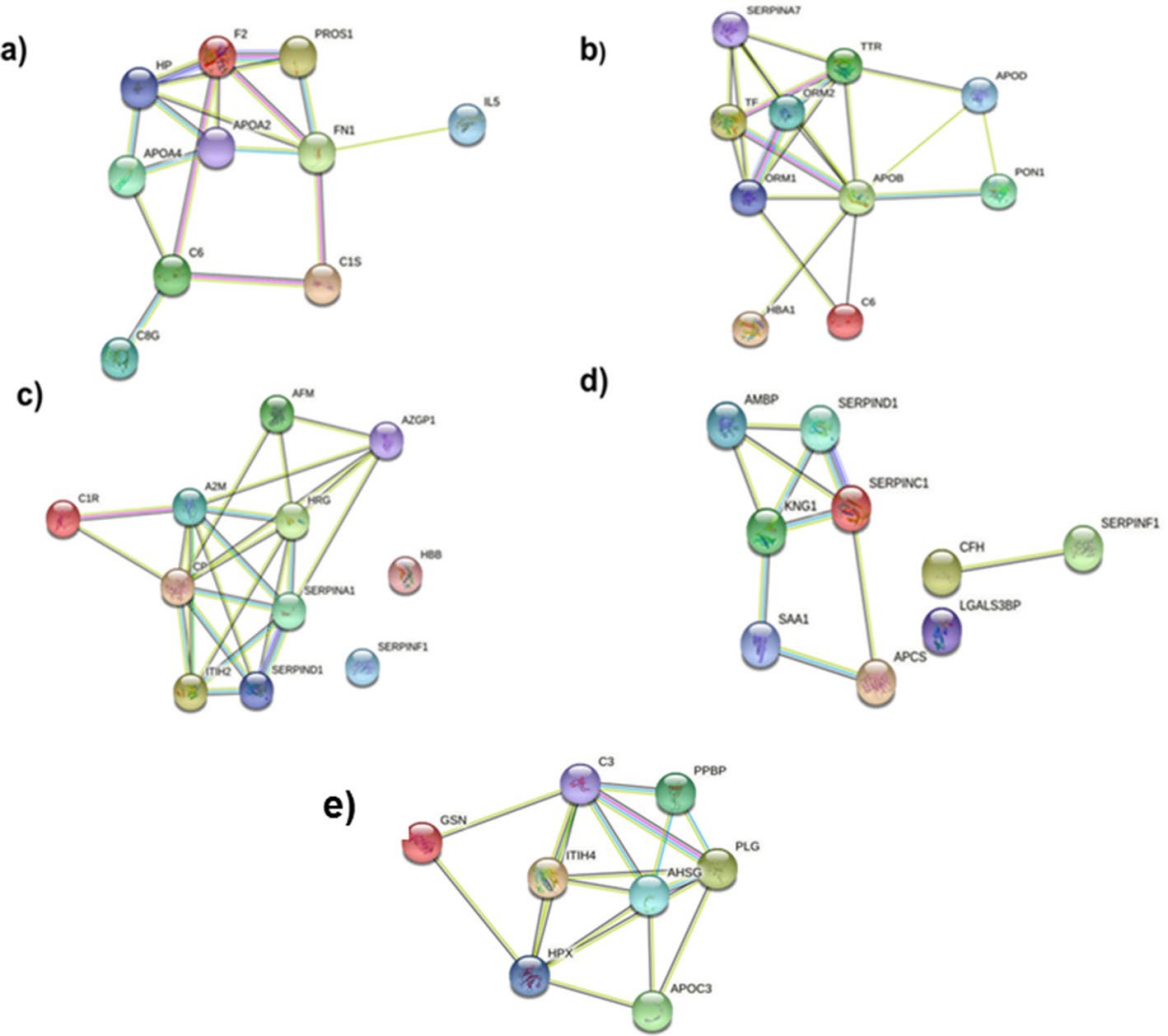
String analysis of both common and exclusive proteins in both GA and AA genotypes. (**a**) Exclusive up regulated proteins in GA genotype. (**b**) Exclusive up regulated proteins in AA genotype (**c**) Exclusive down regulated proteins in GA genotype. (**d**) Exclusive down regulated proteins in AA genotype. (**e**) Common proteins in both GA and AA as compared to wild type GG genotype.

Six proteins (PPBP, PLG, GSN, APOC3, AHSG, and IGKV3-11) showed a decreased differential expression in AA genotype, and increased in GA genotype in comparison to GG. Molecular function analysis showed that these proteins were involved in neutrophil activation, blood coagulation, actin modulation, hepatic inhibition of triglyceride-rich particles, suppressing of arterial calcification, and antigen binding (Fig. 4a,b). The relevant protein to protein interactions among them is presented in Fig. 5e.

Furthermore, the exclusive proteins of both the GA and the AA genotype carriers as compared to the wild GG genotype carrier were also analyzed, and most of them were found to be in the category of protein modifying enzymes, transfer/carrier protein, metabolite interconversion enzyme, protein-binding activity modulator, and defense/immunity proteins. The percentage distribution of each category is given in Fig. 4c and d. Statistical overrepresentation analysis of molecular functions with reference to CAD of both the GA and the AA genotypes showed that phosphatidylcholine-sterol *O*-acyltransferase activator, cholesterol transfer activity, sterol transfer activities, and phosphatidylcholine binding were exclusively up-regulated in the GA, whereas, platelet degranulation, and response to elevated platelet cytosolic Ca^2+^ were exclusively up-regulated in the AA genotype. Similarly, serine-type endopeptidase inhibitor activity and endopeptidase regulation/inhibition were down-regulated in both GA and AA (Fig. 4e and f). String analysis of both up-, and down-regulated exclusive proteins in both genotypes represents strong interactions among them depicted in Fig. 5a–d.

## Discussion

Several biological processes involve different types of biomolecules, and hence a single type of biomolecule may not represent the clear picture at multiple platforms such as genome, transcriptome, proteome, metabolome or ionome^36–38^. Discovery of new biological insight has become challenging and hindered due to difficulties in combining single-omic datasets in a meaningful manner. Therefore, it is important to consider these biological layers as separate elements and also their interaction with one another for more comprehensive understanding of fundamental biological processes.

The Apolipoprotein B gene (*APOB*) has been associated with dyslipidemia and a risk factor of Cardio Vascular Diseases (CVDs), especially the Coronary Artery Disease (CAD). Various genetic determinants of *APOB* are known to be associated with increased LDL-cholesterol level in CAD^39^. However, to understand the complex disease etiology, and for more efficient therapeutic targets, genetic predisposition does not provide a clear picture. To understand the genotype–phenotype relationship, several layers of information through the multiple “Omics” platforms are required. In the present study, we used proteogenomic approach to better understand the disease pathology. This is to the best of our knowledge, the first proteogenomic approach used to study the *rs562338* (G/A) polymorphism of the *APOB* gene in CAD in a Pakistani cohort.

In the current study, majority of the patients were enrolled in the study at the time of admission or after 1 day of their admission to Coronary Care Unit. Data related to their drug regime showed that ~ 9% patients were taking antihypertensive drugs while < 20% patients were prescribed to take statins. With a half-life of 1–3 h, the statin must be administered in multiple doses in order to produce an effect in the patients^40^. Therefore, it was understood that the initial level of statin in CAD patient’s serum could not significantly influence the cholesterol metabolism related pathways. In the first part of genotyping of *rs562338* (G/A) of the *APOB*, we found the frequency of minor allele of *rs562338*-A to be 7% in our patient cohort, which was high in comparison to 1.1% in the HapMap-HCB (Han Chinese in Beijing), and < 1% in the Han Chinese population^13,41^. These differences were due to significant ethnic diversity between the two populations. We found strong association of genotype *rs562338*-AA with increased risk of CAD (CAD cases versus healthy controls: odds ratio (OR) = 4: 95% CI 1.9–16.7;* p* = 0.04). These findings are in agreement with multiple studies in the American and European populations, which showed strong association of *rs562338* polymorphism with higher levels of LDL-cholesterol, and in turn with an increased risk of CAD^42,43^.

In the second step, using label free proteomics, we analyzed differential expression of significant serum proteins from all three genotypes (GG, GA, and AA) in the CAD patients. The purpose of this strategy was to analyze the influence of these genotypes of *APOB* gene on patient serum proteome. In common proteins comparison, three acute phase proteins^44,45^ (ITIH4, HPX, and C3) were found to be differentially down-regulated in GA as compared to AA, with reference to wild type GG genotype. ITIH4 has been reported to be a putative anti- inflammatory marker in ischemic stroke^46^, and several types of cancers^47–49^. HPX is a 60-kDa plasma glycoprotein which represents the primary line of defense against heme-related oxidative stress, and toxicity^50^. The HPX molecule acts as a heme-specific carrier from the bloodstream to the liver and excess heme may be detrimental to tissues by mediating oxidative and inflammatory injuries^51,52^. HPX is also known to inhibit the LDL oxidation, and hence reduce atherogenesis^53^. In our study, the levels of ITIH4 and HPX were up-regulated in the AA genotype and down-regulated in GA genotype as compared to GG genotype. These results showed less anti-inflammatory activity in AA in contrast to GG genotype. C3, the complement protein, secreted by liver and adipose tissues, is the central component of the complement system. Our findings are in agreement with several studies which reported that complement C3 as possible biomarker of cardio-metabolic diseases, and insulin resistance^54–56^.

Out of six common up-regulated proteins in GA genotype, the highest fold change was observed in PLG, PPBP and APOC3. PLG (plasminogen protein) plays a pivotal role in fibrinolysis and wound healing. This protein generates the active enzyme plasmin which is essential for the dissolution of blood clots, and is important in wound healing^57^. Its deficiency may result in increased risk of thrombosis^58^. In agreement with our findings, Folsom et al., also found a positive association between PLG and the risk of cardiovascular diseases^59^. Pro-platelet basic protein (PPBP) or chemokine (C-X-C motif) ligand 7 (CXCL7) is a small cytokine of CXC chemokine family. It is released in large amount from activated platelets in response to vascular injury^60^. It stimulates various processes, including mitogenesis, glucose metabolism, and the synthesis of extracellular matrix and is a plasminogen activator^61,62^. In Thai hyperlipidemia patients, Maneerat et al., found strong correlation of PPBB with the risk of CHD development^63^. In the present study, we also found the up-regulation of ApoC3 in GA. ApoC3, also known as aplolipoprotein C3, is a carrier/transporter protein found on the surface of triglyceride rich lipoproteins (TRLs), such as chylomicrons, VLDL, and remnant cholesterol^64^. Recent evidences have suggested that it promotes the vascular inflammation and TRLs mediated atherogenicity. Furthermore, dysfunctional ApoC3 is associated with lower levels of plasma triglycerides and a reduced risk of CHD^65,66^. Our data suggest that the GA genotype is more prone to TRLs mediated atherogenicity, as compared to the AA genotype.

In the current study, we also found some proteins exclusively identified in CAD patients with GA and AA genotypes with reference to patient with the GG genotypes (Fig. 4a,c). In the exclusive protein comparison of GA/GG genotype, the GO annotation shows an up-regulation of activities, including phosphatidylcholine-sterol *O*-acyltransferase activator activity, cholesterol transfer activity, sterol transfer activity and phosphatidylcholine binding (Fig. 4b). All of these activities were involved in the regulation and uptake of cholesterol, and reverse cholesterol transport. High level of phosphatidylcholine-sterol *O*-acyltransferase or LCAT might be associated with decrease in LDL particle size, and increase in TRL markers in CVD patients^67,68^. Our data suggest that the GA genotype has high LCAT activity, as compared to the GG genotype and may have less atherogenic risk in terms of LCAT related genes. Similarly, the up-regulated GO annotated functions in AA genotype include, platelets degranulation and their response to elevated level of cytosolic Ca^2+^. Both of these responses are involved in platelets activation which play important role in the pathophysiology of CVDs. Because these proteins are implied in thrombus formation after atheroma plaque rupture^69,70^. Furthermore, the serine-type endopeptidase inhibitor activity was found to be down-regulated in both GA and AA genotypes. Serine proteases are key components of the inflammatory response, and play a major role in the body's defense mechanisms, as well as vascular homeostasis and tissue remodeling^71^. These proteins are produced either through in coagulation cascade or discharged from activated leukocytes and mast cells. Multiple studies found that leukocyte activation in several conditions, including infection, hypertension, hyperlipidemia, hyperglycemia, obesity, and atherosclerosis, are associated with increased CVD risks^72–75^. Their down-regulation in our study suggests protection from CAD.

Overall, the proteomic analysis showed significant up-regulation of proteins involved in pathways related to the pathogenesis of CAD, such as cholesterol metabolism, in AA genotype as compared to the GG genotype. This finding is in parallel to the genomic association of AA genotype with the risk of CAD. Furthermore, these results are compatible with the findings of the biochemical analysis of our studied metabolites. Such that we found high levels of triglycerides (significant), cholesterol (significant), and LDL (significant); and low levels of HDL (significant) as compared to the control group. This represent disturbances in cholesterol related pathways.

In the present study, a strong association of the rs*562338*-AA genotype of *APOB* gene with CAD risk in Pakistani population was found. However, there are certain limitations of the study. *APOB* is a large gene with 43 kb size, observing the effect of multiple polymorphisms of this gene on CAD proteome was out of scope of the objectives of the current research work. The present study observed the effect of single SNP, however the chances of effect of other SNPs on the proteomics of the presented patient’s serum samples cannot be excluded. Further, CAD is a complex metabolic condition in which multiple factors are responsible for the pathogenesis of the disease. However, current study is only presenting the effect of SNP on the proteomics of the CAD patient’s serum samples, the metabolomics profiling of the same samples may reveal more detailed picture of the perturbations observed in the molecular pathways. Further, there are some specific limitations related to the subjects of the study. Such as, recruitment of controls was done on the basis of baseline biochemical parameters, and previous history, only. Moreover, the data of other clinical parameters like ejection fraction, and cardiac biomarkers were not collected, and therefore may have any impact on the results. Due to limited sample size, the study has a low statistical power, and less frequency of *APOB* rs*562338*-AA genotypes. A large size population-based study is recommended to increase the statistical power and to confirm any ethnic differences of this polymorphism.

## Conclusion

In summary, we have found a strong association of the rs*562338*-AA genotype (recessive model) of *APOB* gene with CAD risk in Pakistani population. Similarly, in the serum proteomic analysis the AA genotype of rs*562338* (G/A) polymorphism is more actively involved in CAD relevant pathways, as compared to the GG genotype. This genotypic-phenotypic study provides a better understanding of CAD prevalence in local populations. In future, such studies need to be conducted on a large scale on different sub-population group to validate the effect of multiple genetic determinants on complex and multifactorial diseases occurrence, such as CVDs. Furthermore “proteogenomics” approach is recommended to better understand the disease pathology, and to pave the way for more efficient and personalized therapeutic interventions.

## Supplementary Information

Supplementary Information.

## References

[1] Masson W (2020). Role of non-statin lipid-lowering therapy in coronary atherosclerosis regression: A meta-analysis and meta-regression. Lipids Health Dis..

[2] Zdravkovic S (2002). Heritability of death from coronary heart disease: A 36 year follow up of 20 966 swedish twins. J Inter. Med..

[3] Fischer M (2005). Distinct heritable patterns of angiographic coronary artery disease in families with myocardial infarction. Circulation.

[4] van der Harst P, Verweij N (2018). Identification of 64 novel genetic loci provides an expanded view on the genetic architecture of coronary artery disease. Circ. Res..

[5] Teslovich TM (2010). Biological, clinical and population relevance of 95 loci for blood lipids. Nature.

[6] Aulchenko YS (2009). Loci influencing lipid levels and coronary heart disease risk in 16 european population cohorts. Nat. Genet..

[7] Kathiresan S (2009). Common variants at 30 loci contribute to polygenic dyslipidemia. Nat. Genet..

[8] Kettunen J (2012). Genome-wide association study identifies multiple loci influencing human serum metabolite levels. Nat. Genet..

[9] Langlois M (2018). European atherosclerosis society (eas) and the european federation of clinical chemistry and laboratory medicine (eflm) joint consensus initiative. Quantifying atherogenic lipoproteins: Current and future challenges in the era of personalized medicine and very low concentrations of ldl cholesterol. A consensus statement from eas and eflm. Clin. Chem..

[10] Sharifi M, Futema M, Nair D, Humphries SE (2017). Genetic architecture of familial hypercholesterolaemia. Curr. Cardiol. Rep..

[11] Defesche JC (2017). Familial hypercholesterolaemia. Nat. Rev. Dis. Primers..

[12] Jeemon P, Pettigrew K, Sainsbury C, Prabhakaran D, Padmanabhan S (2011). Implications of discoveries from genome-wide association studies in current cardiovascular practice. World J. Cardiol..

[13] Gu Q (2017). Association between polymorphisms in the apob gene and hyperlipidemia in the chinese yugur population. Braz. J. Med. Biol. Res..

[14] Mach F (2020). 2019 esc/eas guidelines for the management of dyslipidaemias: Lipid modification to reduce cardiovascular risk. Eur. Heart J..

[15] Kessler T, Vilne B, Schunkert H (2016). The impact of genome wide association studies on the pathophysiology and therapy of cardiovascular disease. EMBO Mol. Med..

[16] Brænne I (2015). Prediction of causal candidate genes in coronary artery disease loci. Arteroscler. Thromb. Vasc. Biol..

[17] Ritchie MD, Holzinger ER, Li R, Pendergrass SA, Kim D (2015). Methods of integrating data to uncover genotype–phenotype interactions. Nat. Rev. Genet..

[18] Hartiala J (2017). The genetic architecture of coronary artery disease: Current knowledge and future opportunities. Curr. Atheroscler. Rep..

[19] Zhao Y (2019). Multi-omics integration reveals molecular networks and regulators of psoriasis. BMC Sys. Biol..

[20] Hasin Y, Seldin M, Lusis A (2017). Multi-omics approaches to disease. Gen. Biol..

[21] Arneson D (2017). Multidimensional integrative genomics approaches to dissecting cardiovascular disease. Front Cardiovasc. Med..

[22] Manzoni C (2018). Genome, transcriptome and proteome: The rise of omics data and their integration in biomedical sciences. Brief Bioinform..

[23] Faulkner S, Dun MD, Hondermarck H (2015). Proteogenomics: Emergence and promise. Cell. Mol. Life Sci..

[24] Thomas PG (2005). Recommendations for blood pressure measurement in humans and experimental animals: part 1: Blood pressure measurement in humans: A statement for professionals from the Subcommittee of Professional and Public Education of the American Heart Association Council on High Blood Pressure Research. Circulation.

[25] Marc-Andr C (2011). Scientific statement from the American Heart Association. Circulation.

[26] McKiernan H, Danielson P (2017). Molecular Diagnostics.

[27] Alyethodi RR (2018). T-arms pcr genotyping of snp rs445709131 using thermostable strand displacement polymerase. BMC Res. Note.

[28] Bradford M (1979). Nothofagus. J. Forest..

[29] Mirza MR (2012). A novel strategy for phosphopeptide enrichment using lanthanide phosphate co-precipitation. Anal. Bioanal. Chem..

[30] Beltran-Camacho L (2020). Identification of the initial molecular changes in response to circulating angiogenic cells-mediated therapy in critical limb ischemia. Stem Cell Res. Ther..

[31] Cox J (2011). Andromeda: A peptide search engine integrated into the maxquant environment. J. Proteome. Res..

[32] Cox J, Matthias M (2008). MaxQuant enables high peptide identification rates, individualized p.p.b.-range mass accuracies and proteome-wide protein quantification. Nat. Biotech..

[33] Christian LA (2010). Quantitative proteomics reveals subset-specific viral recognition in dendritic cells. Immunity.

[34] Green GH, Diggle PJ (2007). On the operational characteristics of the Benjamini and Hochberg false discovery rate procedure. Stat. Appl. Genet. Mol. Biol..

[35] Szklarczyk D (2010). The string database in 2011: Functional interaction networks of proteins, globally integrated and scored. Nucleic Acids Res..

[36] Kurakin A (2009). Scale-free flow of life: On the biology, economics, and physics of the cell. Theor. Biol. Medical. Model..

[37] Gutteridge A (2010). Nutrient control of eukaryote cell growth: A systems biology study in yeast. BMC Biol..

[38] Bensimon A, Heck AJ, Aebersold R (2012). Mass spectrometry-based proteomics and network biology. Annu. Rev. Biochem..

[39] Willer CJ (2008). Newly identified loci that influence lipid concentrations and risk of coronary artery disease. Nat. Genet..

[40] Katzung BG (2012). Basic and Clinical Pharmacology, (12th edition).

[41] Lettre G (2011). Genome-wide association study of coronary heart disease and its risk factors in 8,090 african americans: The nhlbi care project. PLoS Genet..

[42] Choi-Miura NH (2012). Quantitative measurement of the novel human plasma protein, ihrp, by sandwich elisa. Biol. Pharm. Bull..

[43] Sandhu MS (2008). Ldl-cholesterol concentrations: A genome-wide association study. The Lancet.

[44] Piñeiro M (1999). ITIH4 serum concentration increases during acute-phase processes in human patients and is up-regulated by interleukin-6 in hepatocarcinoma hepg2 cells. Biochem. Biophys. Res. Commun..

[45] Kashyap RS (2009). Inter-α-trypsin inhibitor heavy chain 4 is a novel marker of acute ischemic stroke. Clin. Chim. Acta..

[46] Villanueva J (2006). Differential exoprotease activities confer tumor-specific serum peptidome patterns. J. Clin. Investig..

[47] van Winden AW (2010). Serum degradome markers for the detection of breast cancer. J. Proteome. Res..

[48] Song J (2006). Quantification of fragments of human serum inter-α-trypsin inhibitor heavy chain 4 by a surface-enhanced laser desorption/ionization-based immunoassay. Clin. Chem..

[49] Tolosano E, Altruda F (2002). Hemopexin: Structure, function, and regulation. DNA Cell Biol..

[50] Kumar S, Bandyopadhyay U (2005). Free heme toxicity and its detoxification systems in human. Toxicol. Lett..

[51] Tolosano E, Fagoonee S, Morello N, Vinchi F, Fiorito V (2010). Heme scavenging and the other facets of hemopexin. Antioxid. Redox Signal..

[52] Balla J (2005). Heme, heme oxygenase and ferritin in vascular endothelial cell injury. Mol. Nutr. Food Res..

[53] Vaisar T (2007). Myeloperoxidase and inflammatory proteins: Pathways for generating dysfunctional high-density lipoprotein in humans. Curr. Atheroscler. Rep..

[54] Bratti LDOS (2017). Complement component 3 (c3) as a biomarker for insulin resistance after bariatric surgery. Clin. Biochem..

[55] Ahmad RMH, Al-Domi HA (2017). Complement 3 serum levels as a pro-inflammatory biomarker for insulin resistance in obesity. Diabetes Metab. Syndr..

[56] Ursini F, Abenavoli L (2018). The emerging role of complement c3 as a biomarker of insulin resistance and cardiometabolic diseases: Preclinical and clinical evidence. Rev. Recent Clin. Trials..

[57] Martin-Fernandez L (2016). The unravelling of the genetic architecture of plasminogen deficiency and its relation to thrombotic disease. Sci. Rep..

[58] Mehta R, Shapiro A (2008). Plasminogen deficiency. Haemophilia.

[59] Folsom AR (2000). Fibrinolytic factors and atherothrombotic events: Epidemiological evidence. Ann. Med..

[60] Stankiewicz AM (2014). Social stress increases expression of hemoglobin genes in mouse prefrontal cortex. BMC Neurosci..

[61] Hristov M (2007). Importance of cxc chemokine receptor 2 in the homing of human peripheral blood endothelial progenitor cells to sites of arterial injury. Circ. Res..

[62] Majumdar S, Gonder D, Koutsis B, Poncz M (1991). Characterization of the human beta-thromboglobulin gene: Comparison with the gene for platelet factor 4. J. Biol. Chem..

[63] Maneerat Y, Prasongsukarn K, Benjathummarak S, Dechkhajorn W (2017). Ppbp and defa1/defa3 genes in hyperlipidaemia as feasible synergistic inflammatory biomarkers for coronary heart disease. Lipids Health Dis..

[64] Rocha NA, East C, Zhang J, McCullough PA (2017). Apociii as a cardiovascular risk factor and modulation by the novel lipid-lowering agent volanesorsen. Curr. Atheroscler. Rep..

[65] TGH Group (2014). Working group of the exome sequencing project, national heart, lung, and blood institute, Loss-of-function mutations in apoc3, triglycerides, and coronary disease. N. Engl. J. Med..

[66] Jørgensen AB, Frikke-Schmidt R, Nordestgaard BG, Tybjærg-Hansen A (2014). Loss-of-function mutations in apoc3 and risk of ischemic vascular disease. N. Engl. J. Med..

[67] Tani S, Takahashi A, Nagao K, Hirayama A (2016). Association of lecithin–cholesterol acyltransferase activity measured as a serum cholesterol esterification rate and low-density lipoprotein heterogeneity with cardiovascular risk: A cross-sectional study. Heart Vessels.

[68] Yokoyama K, Tani S, Matsuo R, Matsumoto N (2018). Association of lecithin-cholesterol acyltransferase activity and low-density lipoprotein heterogeneity with atherosclerotic cardiovascular disease risk: A longitudinal pilot study. BMC Cardiovasc. Disord..

[69] Ahmed M, Jadhav A, Hassan A, Meng QH (2012). Acute phase reactants as novel predictors of cardiovascular disease. Int. Sch. Res. Notices..

[70] Vélez P, García A (2015). Platelet proteomics in cardiovascular diseases. Transl. Proteom..

[71] Sharony R (2010). Protein targets of inflammatory serine proteases and cardiovascular disease. J. Inflamm..

[72] Granger DN, Vowinkel T, Petnehazy T (2004). Modulation of the inflammatory response in cardiovascular disease. Hypertension.

[73] Mügge A (1991). Mechanisms of contraction induced by human leukocytes in normal and atherosclerotic arteries. Circ. Res..

[74] Marcondes S, Antunes E (2005). The plasma and tissue kininogen-kallikrein-kinin system: Role in the cardiovascular system. Curr. Med. Chem. Cardiovasc. Hematol. Agents..

[75] Pejler G, Rönnberg E, Waern I, Wernersson S (2010). Mast cell proteases: Multifaceted regulators of inflammatory disease. Blood.

